# Projected Impacts of Climate Change on Environmental Suitability for Malaria Transmission in West Africa

**DOI:** 10.1289/ehp.1206174

**Published:** 2013-09-16

**Authors:** Teresa K. Yamana, Elfatih A.B. Eltahir

**Affiliations:** Ralph M. Parsons Laboratory, Department of Civil and Environmental Engineering, Massachusetts Institute of Technology, Cambridge, Massachusetts, USA

## Abstract

Background: Climate change is expected to affect the distribution of environmental suitability for malaria transmission by altering temperature and rainfall patterns; however, the local and global impacts of climate change on malaria transmission are uncertain.

Objective: We assessed the effect of climate change on malaria transmission in West Africa.

Methods: We coupled a detailed mechanistic hydrology and entomology model with climate projections from general circulation models (GCMs) to predict changes in vectorial capacity, an indication of the risk of human malaria infections, resulting from changes in the availability of mosquito breeding sites and temperature-dependent development rates. Because there is strong disagreement in climate predictions from different GCMs, we focused on the GCM projections that produced the best and worst conditions for malaria transmission in each zone of the study area.

Results: Simulation-based estimates suggest that in the desert fringes of the Sahara, vectorial capacity would increase under the worst-case scenario, but not enough to sustain transmission. In the transitional zone of the Sahel, climate change is predicted to decrease vectorial capacity. In the wetter regions to the south, our estimates suggest an increase in vectorial capacity under all scenarios. However, because malaria is already highly endemic among human populations in these regions, we expect that changes in malaria incidence would be small.

Conclusion: Our findings highlight the importance of rainfall in shaping the impact of climate change on malaria transmission in future climates. Even under the GCM predictions most conducive to malaria transmission, we do not expect to see a significant increase in malaria prevalence in this region.

Citation: Yamana TK, Eltahir EA. 2013. Projected impacts of climate change on environmental suitability for malaria transmission in West Africa. Environ Health Perspect 121:1179–1186; http://dx.doi.org/10.1289/ehp.1206174

## Introduction

The response of malaria transmission to climate change has been the subject of research and intense debate since the mid-1990s, and it has been investigated using both biological/mechanistic models and statistical models (Parham and Michael 2010; Rogers and Randolph 2000). Although early studies reported predictions of a widespread increase in malaria transmission (Martens P et al. 1999; Martens W et al. 1995; Martin and Lefebvre 1995; Tanser et al. 2003), more recent studies suggest a shift in distribution rather than a large net increase (Ermert et al. 2012; Lafferty 2009; Thomas et al. 2004).

Previous studies on this topic in West Africa have been limited by the relatively crude representation of processes dependent on rainfall in malaria models, as well as by the great uncertainty in climate change projections in this region. Although the relationships between temperature and malaria transmission are relatively well understood, modeling methods that have been used up to now to estimate the effect of climate change on malaria transmission are limited in their ability to address the effects of changes in rainfall. The primary malaria vectors in Africa, *Anopheles gambiae* sensu lato and *Anopheles funestus,* breed primarily in pools of water formed from rainfall. Few malaria models attempt to model the causal relationships between rainfall and mosquito breeding sites, relying instead on rules for minimum threshold values of rainfall required for malaria transmission to occur (Craig et al. 1999; Martens et al. 1999), with some models including an upper threshold of rainfall above which additional rainfall is assumed to decrease mosquito density (Parham and Michael 2010). Shaman et al. (2002) and Porphyre et al. (2005) used hydrological models to link rainfall to the abundance of *Culex* and *Aedes* mosquitoes, which breed in floodwaters and serve as the primary vectors for several arboviruses. Montosi et al. (2012) used an ecohydrological model as well as a simplified linear model to calculate soil water content, which was then used to model malaria incidence. The processes by which rainfall is diverted into pools suitable for *Anopheles* breeding are strongly dependent on the frequency, intensity, and duration of rainfall events in addition to site-specific topographical features, soil characteristics, and vegetation cover. The persistence of these pools depend on evaporation and infiltration rates; pools that dry out before adult mosquitoes emerge from eggs are not viable breeding sites.

Here, we bridge the gap between rainfall and corresponding mosquito abundances using the Hydrology, Entomology, and Malaria Transmission Simulator (HYDREMATS) (Bomblies et al. 2008). By mechanistically translating rainfall into water pools, we can simulate the effects of projected changes in climate on malaria transmission in West Africa. In addition, we address the high uncertainty of climate predictions in this region by estimating the impact of changes in rainfall over the full range predicted by current climate models.

## Methods

*Model description*. We performed data simulations to study the impacts of climate change on environmental suitability for malaria transmission in West Africa using the HYDREMATS model developed by Bomblies et al. (2008), which has been used in other recent studies in West Africa (Bomblies 2012; Bomblies and Eltahir 2010; Bomblies et al. 2009; Gianotti et al. 2009; Yamana and Eltahir 2010, 2011). Detailed information about HYDREMATS has been reported previously (Bomblies et al. 2008). In brief, the model is a physics-based hydrology model coupled with an individual-based entomology model that is run at a spatial resolution of 10 m with a 1-hr time step (see Supplemental Material, Figure S1). The hydrology component explicitly represents water pools available as breeding sites to anopheline mosquitoes by simulating the flow of rainfall into topographical low points and water loss due to evaporation and infiltration. The temperature of each water pool is computed by solving a system of energy balance and heat transfer equations (Bomblies et al. 2008).

The HYDREMATS entomology component simulates individual mosquito and human agents (see Supplemental Material, Figure S1). Human agents are assumed to be immobile and are assigned to village residences because malaria transmission in this region occurs primarily at night when humans are indoors (Service 1993). Mosquito agents have a probabilistic response to their environment based on a prescribed set of rules governing dispersal and discrete events including feeding, resting, egg-laying, and death (see Supplemental Material, Figure S2, Table S1). The model tracks the location, infective status, and reproductive status of each female mosquito through time.

The aquatic stage of mosquitoes is simulated in water pools. When an adult mosquito in the ovipositing stage encounters a water pool, the probability that she will lay eggs varies depending on water depth. The model assumes that eggs in each pool progress through four larval stages and a pupal stage before emerging as adults at a temperature-dependent rate developed by Depinay et al. (2004) [see Supplemental Material, Methods: Development rate of aquatic-stage mosquitoes (p. 5) and Table S2]. Aquatic stage mosquitoes contained in a pool that dries out are killed, reflecting the importance of pool persistence to larval development.

Whereas previous studies using HYDREMATS focused on *Anopheles gambiae* sensu lato mosquitoes, here we also consider *A. funestus*, another important vector in the wetter parts of West Africa. The primary difference between the two types of mosquitoes is their breeding preference: Members of the *A. gambiae* complex breed in small, temporary pools, and *A. funestus* breeds in larger, more persistent water bodies. Both types of pools are modeled in HYDREMATS, and we do not currently distinguish between species of *Anopheles* mosquitoes. The entomological parameters of the model are tuned using data for *A. gambiae* because this complex has been studied much more extensively (Coetzee and Fontenille 2004). Moreover, we do not expect parameter values specific to *A. funestus* to be significantly different because the two types of mosquitoes have similar adult survival and dispersal behavior (Midega et al. 2007) and both are primarily nocturnal, endophagic, and anthrophilic (Horsfall 1943).

The model output most relevant to our study is the vectorial capacity (*VC*), which is a measure of environmental suitability for malaria transmission defined as the average number of human inoculations of a parasite originating from a single case of malaria if all vectors biting the original case were to become infected (Garrett-Jones and Grab 1964). We compute *VC* using the following set of equations:

*VC* = *ma*^2^*D,* [1]

where *m* is the number of female mosquitoes per human simulated by HYDREMATS, *a* is the average number of bites taken by each mosquito per time step, and *D* is the expected duration of infective life of the mosquito (in days). A constant biting rate (*a* = 0.2) is assumed, consistent with observations in this region (Garrett-Jones and Shidrawi 1969).

*D* is defined as the number of days an average mosquito will be infective and is a function of temperature, maximized at 28°C. *D* is given by the following equation:

*D* = *p^EIP^*/–ln(*p*), [2]

where *p* is the daily survival probability of the mosquito, and *EIP* is the extrinsic incubation period, defined as the number of days *Plasmodium falciparum* must be present within the mosquito before it can be transmitted to humans.

The survival of mosquitoes is given by the equation:

*p* = exp[–1/(–4.4 + 1.31*T* – 0.03*T* ^2^)], [3]

where *T* is the daily average air temperature in degrees Celsius (Martens 1997). This function gives maximum longevity in the range of 20–25°C, and severe mortality at temperatures < 10°C and > 35°C.

*EIP* is given by the equation:

*EIP* = 111/(*T* – 16), [4]

where *T* is the daily average air temperature in degrees Celsius (Detinova 1962). Malaria transmission can only occur when the mosquito lifespan exceeds the *EIP*. We also tested the sensitivity of our results to an alternate formulation for *EIP* (Paaijmans et al. 2009)[see Supplemental Material, Methods: Alternate *EIP* formulation (p. 6) and Figure S3].

**Figure 1:**
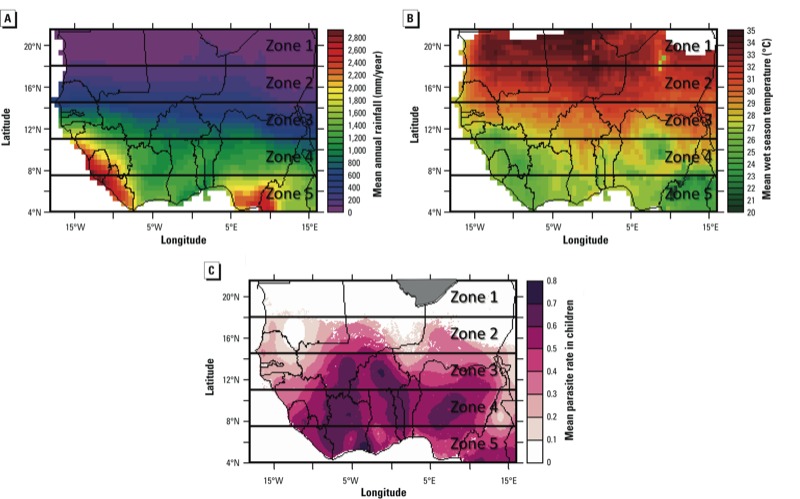
Baseline climate and malaria transmission conditions in West Africa. Zones 1–5 correspond roughly to the following ecoclimate zones: Zone 1, Sahelo-Sahara; Zone 2, Sahel; Zone 3, Soudan; Zone 4, Soudano-Guinean; and Zone 5, Guinea Coast (Nicholson 1993). (*A*) Mean annual rainfall (mm/year) from CRU, 1980–1999 [data from Mitchell and Jones (2005)]. (*B*) Mean surface air temperature (°C) during the wet season from CRU, 1980–1999 [data from Mitchell and Jones (2005)]. (*C*) Mean parasite rate in children 2–10 years of age in 2007 estimated by the Malaria Atlas Project (Hay et al. 2009); white areas over land indicate unstable malaria transmission, and the gray area in Zone 1 indicates no malaria risk.

*Study area.* The climate of West Africa is distinctively characterized by strong north to south gradients in both temperature and rainfall (Figure 1A,B). The climate is highly seasonal, dominated by the West African monsoon. We focused on the region bounded by 4°N and 21.5°N, and 18°W and 16°E, which we divided into five subregions (Zones 1–5 in Figure 1), corresponding roughly to the following ecoclimate zones, respectively: Sahelo-Sahara, Sahel, Soudan, Soudano-Guinean, and Guinea Coast (Nicholson 1993).

The baseline period for this study was 1980–1999, in keeping with the Intergovernmental Panel on Climate Change’s Fourth Assessment Report (IPCC 2007; Solomon et al. 2007). The mean annual rainfall and wet-season temperature for each zone were calculated for the baseline period using standard climate data from Climatic Research Unit time-series version 3.1 (CRU) data (Mitchell and Jones 2005) (Table 1).

**Table 1:** Characteristics of the study zones.

Zone	Ecoclimate zone^*a*^	Annual rainfall 1980–1999 (mm)^*b*^	Mean wet season temperature 1980–1999 (°C)^*b*^	Malaria transmission class 2007^*c*^
1	Sahelo-Sahara	52	32.2	Unstable
2	Sahel	223	31.3	Unstable/moderate stable
3	Soudan	715	28.9	Moderate/intense
4	Soudano-Guinea	1,286	26.8	Moderate/intense
5	Guinea Coast	1,743	25.7	Intense
^***a***^Data from Nicholson 1993. ^***b***^Data from Mitchell and Jones 2005. ^***c***^Malaria endemicity class based on criteria outlined by Hay et al. (2008) and calculated from mean parasite rate in children 2–10 years of age in 2007 estimated by the Malaria Atlas Project (Hay et al. 2009).				

We focused on this West African region because of its significant malaria burden. The spatial distribution of parasite rate in children 2–10 years of age in 2007 estimated by the Malaria Atlas Project (Hay et al. 2009) indicates that malaria burden increases roughly from north to south (Figure 1C). Using the malaria endemicity classification proposed by Hay et al. (2008), Zone 1 experiences unstable transmission, Zone 2 is divided roughly equally between unstable and moderate stable transmission, Zone 3 is a mixture of moderate and intense stable transmission, and Zones 4 and 5 are primarily areas of intense transmission (Table 1). Malaria transmission in regions where transmission is classified as unstable is especially sensitive to effects of climate on vectorial capacity because human populations in these areas have little or no acquired immunity and the infrastructure for malaria control is likely to be limited.

*Design of data simulations: baseline climate*. The first step in estimating the potential impacts of climate change on environmental suitability for malaria transmission was to establish vectorial capacity under baseline conditions using HYDREMATS and current climate data. HYDREMATS is a very fine-resolution model that runs on the village scale. Although this resolution allows us to simulate the details of mosquito breeding and malaria transmission, its high computational cost precludes the simulation of large geographic areas. However, West Africa is well known for its pronounced north–south climate gradient (Figure 1A,B), whereas its climate conditions are relatively constant east to west (Eltahir and Gong 1996; Nicholson 1993). We therefore approximated the *VC* for each zone by simulating conditions for a single hypothetical village with climate conditions that are representative of that zone. We conducted a 7-year simulation at each of the five representative locations under baseline climate conditions.

The CRU data set and other monthly precipitation data available for 1980–1999, the baseline period, are of insufficient temporal resolution to be used with HYDREMATS, which requires as an input a rainfall series with an hourly resolution. To represent the role of fine-scale variability of rainfall in the process of formation of breeding pools, we therefore disaggregated the CRU data into an hourly rainfall time series [see Supplemental Material, Methods: Disaggregation of CRU data into hourly rainfall series (p. 8)].

Temperature, wind speed, wind direction, and radiation data were taken from the ERA-Interim data set (Dee et al. 2011). Vegetation and soil properties were taken from the University of Maryland Department of Geography's Global Land Cover Classification database (Hansen 1998) and the Harmonized World Soil Database, version 1.1 (Food and Agriculture Organization 2013). We assumed typical topographical conditions and household locations as observed in Banizoumbou, Niger (Bomblies et al. 2008), that were held constant among zones. [See Supplemental Material, Methods: Summary of data sources (p. 10) and Table S3 for additional information on these data sources.]

*Design of data simulations: future climate*. After establishing baseline conditions, we repeated the simulations using future climate projections as inputs to HYDREMATS. We considered the entire range of predictions from the 19 general circulation models (GCMs) contributing to the A1B emissions scenario of the IPCC’s Fourth Assessment report (IPCC 2007; Solomon et al. 2007). This scenario describes a future characterized by rapid economic growth; decreased heterogeneity among nations through increased interactions, capacity building, and cooperation; and a balance between fossil fuel and alternative energy sources (Solomon et al. 2007). The models differ greatly in their predictions of future climate in West Africa. This disagreement implies that at least some of the GCMs are substantially flawed in their representation of the climate in this region (Christensen et al. 2007; Cook and Vizy 2006). Therefore, we conducted a preliminary analysis to identify the GCMs that would maximize and minimize vectorial capacity in each zone during 2080–2099, under the assumption that the true outcome will fall within the bounds set by these extreme scenarios. As discussed in detail in “Results,” we determined that the GCMs resulting in the wettest and driest climate projections would produce the maximum and minimum estimates of *VC*, respectively.

We conducted four simulations of future *VC* for each zone. First, to highlight the impact of changes in rainfall, we simulated the predicted changes in rainfall only, while keeping baseline values of temperature and all other variables. Two 7-year simulations were conducted for each region, one using the driest outcome predicted by the models and one using the wettest outcome (referred to as dry and wet simulations, respectively). Next, to assess the combined impact of increased temperature and changing rainfall, we repeated the simulations with predicted temperature increases included in addition to changes in precipitation (dry-hot and wet-warm simulations, respectively).

Projected changes in rainfall and temperature between the baseline period (1980–1999) and the future (2080–2099) are provided by the GCM outputs. We assume that climate change will take the form of shifts in the north–south rainfall gradient, consistent with historical changes in rainfall regimes in this region (Bomblies and Eltahir 2010; Irizarry-Ortiz et al. 2003). The 2080–2099 precipitation time series were created by selecting a location directly north (for decreased rainfall scenarios) or south (for increased rainfall scenarios) of the representative village in each zone where the current rainfall is equal to the annual rainfall predicted by a GCM for 2080–2099, and disaggregating using CMORPH [see Supplemental Material, Methods: 2080–2099 Precipitation time series (p. 11)]. The increase in temperature for each zone was represented by adding the mean wet-season temperature increase of the GCM grid cell containing each village to each hourly data point used in the simulation of baseline climate. The remaining model inputs were not changed.

## Results

**Figure 2:**
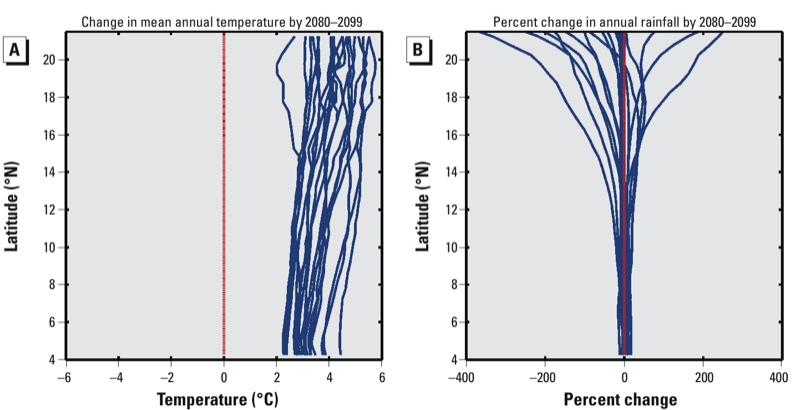
Predicted changes in temperature and rainfall, zonally averaged for each model. Each blue line is the zonally averaged change in temperature (*A*) and rainfall (*B*) predicted between the baseline period (1980–1999) and 2080–2099 by a single SRES A1B GCM, averaged zonally over land points between 18°W and 16°E.

*Analysis of climate predictions from GCMs.* Before conducting our numerical simulations, we analyzed GCM outputs to identify the predictions that would maximize and minimize *VC.* The uncertainty for predicted rainfall is much greater than for predicted temperature; although all of the models predict a temperature increase between 2 and 6°C, the predicted changes in rainfall differ in even their sign and range from a decline of 400% to an increase of 260% (Figure 2). The wide range of possible rainfall outcomes underscores the importance of considering changes in rainfall when assessing future climates.

**Figure 3:**
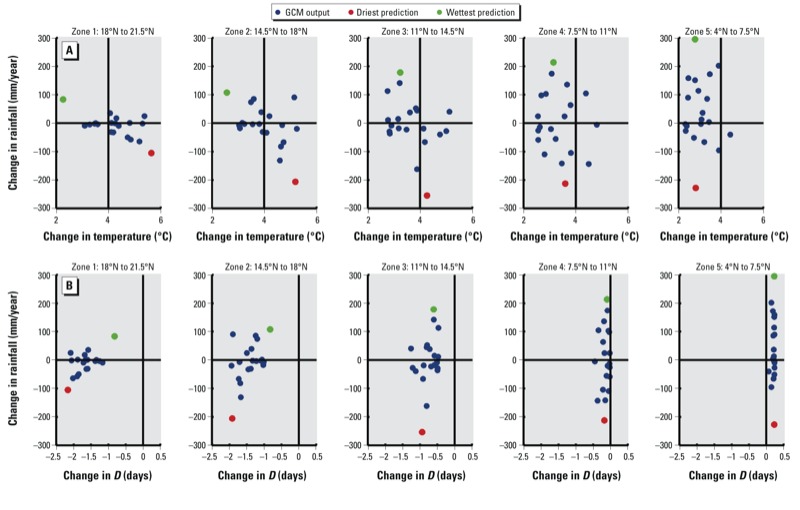
GCM predictions for changes in temperature, rainfall, and expectation of infective life [*D* (in days)]. Each point represents the change in temperature and rainfall (*A*), or the change in the expectation of infective life and rainfall (*B*), predicted by each IPCC AR4 GCM.

The mean change in temperature and precipitation predicted by each GCM is shown in Figure 3A. In Zones 1–3, which are currently drier and warmer than is optimal for malaria transmission, the conditions that would maximize *VC* would be the wettest and coolest prediction, whereas the driest and hottest prediction would minimize *VC*. In these regions, increases in precipitation are associated with less warming because a wetter climate would lead to more evaporative cooling, counteracting some of the warming caused by greenhouse gasses. Similarly, decreases in precipitation are associated with greater warming. This association is less pronounced in Zones 4 and 5 because the relative change in precipitation is much smaller, thus decreasing the impact of the change in evaporative cooling. The changes in the expectation of infective life, *D*, calculated from the predicted changes in temperature from each GCM are shown in Figure 3B. In Zones 1 and 2, the wettest prediction also has the smallest decrease in *D*, and the driest prediction corresponds to the greatest decrease in *D*. In Zones 3, 4, and 5, we assume that the wettest and driest predictions will result in the highest and lowest predictions for vectorial capacity, respectively because the percent change in precipitation between predictions varies more than the percent change in *D* caused by increased temperature. Table 2 summarizes the projected changes in rainfall and temperature corresponding to the two extreme future climate change scenarios for each zone. We did not investigate the accuracy of the climate models, but instead selected the most extreme predictions of climate change, assuming that the resulting simulations would indicate the upper and lower bounds of potential changes in vectorial capacity.

**Table 2:** Changes predicted between 1980–1999 and 2080–2099 by the wettest and driest GCMs for each zone.

Zone	Wettest prediction			Driest prediction		
	GCM^*a*^	Change in rainfall (mm)	Increase in rainy season temperature (°C)	GCM	Change in rainfall (mm)	Increase in rainy season temperature (°C)
1	CCSM3	83	2.3	GFDL-CM2.0	–105	5.6
2	CCSM3	107	2.6	GFDL-CM2.0	–206	5.2
3	ECHO-G	178	3.2	GFDL-CM2.0	–254	4.3
4	ECHO-G	214	3.1	GFDL-CM2.0	–212	3.6
5	GISS EH	295	2.8	MIROC3.2(medres)	–227	2.8
^***a***^Solomon et al. 2007.						

**Figure 4:**
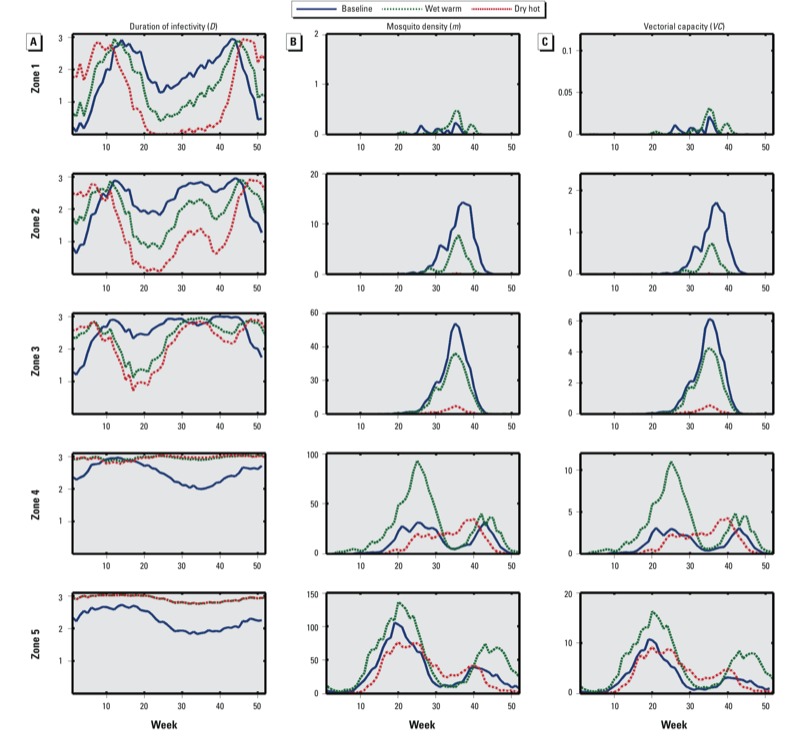
Simulated effects of climate change on expectation of infective life (in days) (*A*), mosquito density (the number of female mosquitoes per human) (*B*), and vectorial capacity (*C*), from Zone 1 through Zone 5. Values shown are weekly averages for baseline simulations (1980–1999) and weekly averages based on wet-warm and dry-hot simulations for 2080–2099.

**Figure 5:**
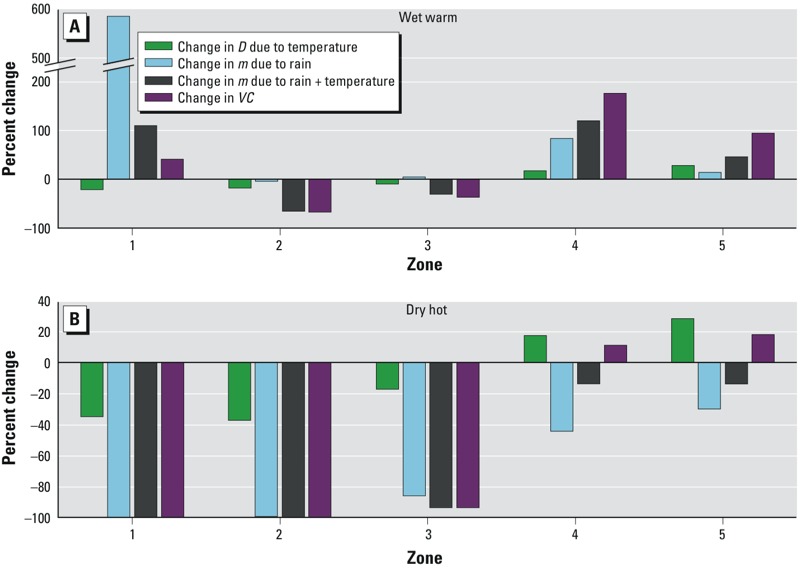
Summary of changes to expectation of infective life (*D*), mosquito density (*m*), and vectorial capacity (*VC*) in response to changes in rainfall and temperature averaged over 7-year simulations for wet-warm (*A*) and dry-hot (*B*) scenarios in climatic Zones 1–5. Note the abbreviated vertical axis in (*A*).

*Simulation results using HYDREMATS*. The results of the simulations were analyzed in terms of the components of the equation for vectorial capacity. Projections of weekly average values over the representative 7-year simulation are shown in Supplemental Material, Figure S4, for simulations that accounted for changing rainfall only, and in Figure 4 for the simulations changing rainfall and temperature. Figure 5 shows the estimated percent change in *D*, *m*, and *VC* averaged over the length of the simulation for each zone.

Expected duration of mosquito infectivity. In the case of changing rainfall only, *D*, the estimated duration of mosquito infectivity, does not change because it depends only on temperature. When we also simulate rising temperatures, *D* increases in Zones 4 and 5 because the temperature in these areas at baseline (1980–1999) is below the optimal temperature for transmission (Figure 4A). The relative changes in *D* in Zones 1, 2, and 3 are highly seasonal (Figure 4A). During the rainy summer months when malaria transmission can occur, the simulated temperature exceeds optimal levels for mosquito survival, resulting in a decrease in *D* and *VC*.

Mosquito density. In general, our simulations predict that increased rainfall will lead to more mosquitoes, although the magnitude of the change varies by region (see Supplemental Material, Figure S4). Relative to baseline values, the greatest predicted increase in *m*, the number of female mosquitoes per human, occurs in Zone 1, where increased rainfall leads to greater persistence of water pools, and in Zone 4, where the earlier onset of the rainy season leads to higher peak values of mosquito populations. When rainfall is predicted to decrease, mosquito populations in all five zones decrease substantially from baseline values, particularly in Zones 1 and 2, which become too dry to sustain mosquito life, and Zone 3 (Figure 4B).

In many cases, the changes in *m* from baseline values predicted by simulations where only rainfall was changed (see Supplemental Material, Figure S4) and simulations where both rainfall and temperature were changed (Figure 4B) is small because *m* depends primarily on rainfall. The potential impact of rising temperatures on mosquito density is more apparent in Figure 5A, where we see that with increased rainfall and warming (wet-warm simulation), the overall effect on Zones 1, 4, and 5 is an increase in *m*; however, in Zones 2 and 3, there is a net decrease in *m*. With warming and decreased rainfall (Figure 5B; dry-hot simulation), high temperatures in Zones 1–3 amplify the estimated effect of decreased rainfall, further decreasing *m*. In Zones 4 and 5, the high temperatures reduce the estimated effect of decreased rainfall, leading to a smaller net reduction in *m*.

We use HYDREMATS to calculate the mosquito density, *m*, which is a function of the number of humans and the total number of mosquitoes. In general, the mosquito population in the village can be described by three different variables: total number of real mosquitoes in the village, total number of simulated mosquitoes in the model, and sampled real mosquitoes in discrete locations captured by light traps. It is not possible to observe the total number of real mosquitoes in the village in order to compare it with the total number of simulated mosquitoes. However, HYDREMATS has been shown to simulate a total number of mosquitoes that mimics the relative differences observed in mosquitoes captured by light traps between wet and dry years (Bomblies et al. 2008) and under different hydrological conditions (Bomblies et al. 2009).

We hold the number of humans and the configuration of residences constant among villages now and in the future, an assumption that affects *m*. This assumption allows us to isolate the impact of climate change on vectorial capacity while neglecting the potential impacts of the human population variability and change in space and time.

Vectorial capacity. As with the density of mosquitoes, in many cases accounting for changes in temperature, in addition to precipitation (i.e., in the wet-warm and dry-hot simulations, Figure 4C), had a relatively small impact on *VC* (see Supplemental Material, Figure S4), which highlights the importance of rainfall in assessing future *VC*. In the wet-warm scenario (Figure 5A), there is an overall increase in *VC* in Zones 1, 4, and 5. In contrast, there is little change in *VC* from baseline in Zones 2 and 3 because the positive effect of increased rainfall on mosquito density is offset by negative effects of higher temperatures on both density and the duration of infectivity. In the dry-hot simulations (Figure 5B), *VC* is reduced to zero in Zones 1 and 2 and substantially decreased in Zone 3, whereas there is a small increase in *VC* in Zones 4 and 5 because the positive effect of warmer temperatures outweighs the decrease in breeding sites with reduced precipitation. However, in almost all cases, the estimated percent change in *m*, which depends primarily on rainfall, is greater than the percent change in *D*, which depends on temperature only, thus adding further support for the importance of rainfall.

Finally, we also simulated the effects of climate change using an alternate formulation for *EIP* developed by Paaijmans et al. (2009) [see Supplemental Material, Results: Alternate EIP formulation (p. 13) and Figures S3 and S5]. Although this formulation led to lower predicted values of *D* and *VC* in all simulations, relative changes between the baseline climate and future climate were similar to those based on the main analyses, except that the dry-hot conditions are predicted to lead to a small decrease of *VC* in Zones 4 and 5 rather than a small increase.

## Discussion

We simulated the effects of projected changes in climate on malaria transmission in West Africa over a range of scenarios predicted by current climate models, and found that the potential impact of changes in rainfall patterns on malaria transmission may be as great as or greater than the potential impact of rising temperatures. However, our findings should be interpreted in light of model assumptions and limitations. We do not consider changes in extreme weather events, which would have an impact on the hydrology and water pool availability of the region. We also do not account for possible shifts in mosquito species, changes to vegetation that may occur as a result of climate change, or changes in nonenvironmental factors that will influence malaria transmission in this region (e.g., malaria control activities, access to health care, improved housing structures, migration, changes in population density or land use). Our study was limited to rural settings where the primary mosquito breeding sites are formed from rainwater. The model was developed and tested in the semi-arid climate characteristic of Zones 1–3; thus it is possible that it does not fully represent some of the hydrological processes of the wetter Zones 4 and 5. Although we present results for an ensemble of future climate projections from AR4 GCMs, future research should evaluate the accuracy of each GCM for simulating past and current climate in the region to determine which predictions are the most plausible.

Our simulations suggest that changes in rainfall can have a significant impact on mosquito populations and vectorial capacity in West Africa, particularly in the northern areas where breeding sites (water pools) currently are a limiting factor. In addition, by comparing the predicted effect of changing rainfall alone to the combined effects of changing rainfall and increasing temperature, we demonstrated that temperature also plays an important role in determining the mosquito density and, thereby, influencing vectorial capacity. Our results stress the need to include rainfall in studies linking climate change and malaria. We also highlight the difficulty in making predictions of future environmental suitability for malaria in this region because the GCMs differ greatly in their rainfall predictions. This also is a problem for projecting other impacts of climate change in Africa, for example, on water supplies (De Wit and Stankiewicz 2006) and food security (Lobell and Burke 2008). All research involving the impacts of changing rainfall in Africa should, therefore, take care in selecting appropriate rainfall predictions.

In the arid and semi-arid regions represented by Zones 1, 2, and 3, our simulations suggest that rising temperatures will move environmental conditions toward, and in some cases beyond, the upper limits tolerated by the *Anopheles* mosquito. However, if rainfall increases, the increased availability of breeding sites will tend to raise *VC*, somewhat offsetting the decreases in *VC* due to increasing temperatures. Under the wettest future climate predicted by an IPCC climate model, our simulations suggest that the fringes of the Sahara desert will experience a small increase in *VC* despite extremely hot temperatures. However, in the Sahel region, the predicted impact of warming temperature dominates, and a decrease in *VC* is predicted even under the wettest future climate scenario.

Our simulations predict that the wetter and cooler Soudano-Guinean and Guinea Coast regions (Zones 4 and 5) will experience an increase in *VC* as a result of warming temperatures, regardless of changes in rainfall. The driest scenarios would lead to only a slight and seasonal increase in *VC*, whereas the wettest scenarios could lead to a doubling or tripling of *VC*. However, malaria transmission in these zones is already classified as intense and stable, and thus these areas would be less sensitive to changes in mosquito ecology and vectorial capacity than areas where malaria transmission is unstable (Hay et al. 2009). Children living in such areas experience many malaria infections in their first years of life, and quickly develop immunity to severe disease (Gupta et al. 1999). Therefore, malaria incidence in these areas is likely to be limited primarily by the number of susceptible individuals within the population, rather than inoculation intensity or vectorial capacity. Consequently, even tripling *VC* would not necessarily lead to a significantly higher burden of malaria (Reiter 2008).

In contrast, Zones 1, 2, and 3 represent areas where malaria is unstable, or seasonally stable with lower intensity, and are therefore more sensitive to changes in *VC*. Even under the wettest conditions predicted by GCMs, our simulations predict that *VC* will decrease in Zones 2 and 3, whereas simulations of the hottest and driest scenarios predict the near elimination of mosquito populations in these zones due to a lack of breeding areas and intolerably hot temperatures. Although a 40% increase in *VC* is predicted in Zone 1 under the wet-warm scenario, vectorial capacity would still be too small to sustain malaria transmission in this zone.

## Conclusions

Our simulations suggest that changes in rainfall will be important in shaping the impact of climate change on malaria transmission, and therefore must be considered in order to accurately project the environmental suitability for malaria transmission in future climates. The disagreement among GCM projections for changes in rainfall makes the future of vectorial capacity in West Africa highly uncertain. However, despite this uncertainty, our analysis suggests that we should not expect increases in malaria transmission due to climate change in areas where transmission is currently unstable or stable at low levels. In addition, although we predict a significant increase in vectorial capacity in the two southern zones of our study area, we do not necessarily expect increases in malaria cases there because these areas already have intense stable transmission and are therefore relatively insensitive to changes in vectorial capacity.

In future work, we plan to analyze the skill of current climate models and select climate projections based on model performance in West Africa, with a focus on regions that we have determined *a priori* to be sensitive to changes in vectorial capacity. In addition, we plan to use the immunology component of HYDREMATS to link changes in climate and vectorial capacity to changes in malaria incidence.

## Supplemental Material

(2.4 MB) PDF
