# Supplementary material for: Projected Impacts of Climate Change on Environmental Suitability for Malaria Transmission in West Africa
Source: Environ Health Perspect. 2013 Sep 16;121(10):1179–86. doi: 10.1289/ehp.1206174 (PMC3801455; doi:10.1289/ehp.1206174)
Supplement: (2.4 MB) PDF [file ehp.1206174.s001.508.pdf]

Environmental Health Perspectives doi:10.1289/ehp.1206174

Supplemental Material

Projected Impacts of Climate Change on Environmental Suitability for Malaria Transmission in West Africa

Teresa K. Yamana and Elfatih A.B. Eltahir

Table of Contents

Figure S1: Schematic of HYDREMATS model....................2

Figure S2: Adult mosquito simulation flow....................3

Table S1: Parameters used with entomology model....................4

Methods: Development rate of aquatic-stage mosquitoes....................5

Table S2: Parameters for development rate of subaquatic mosquitoes....................5

Methods: Alternative *EIP* formulation....................6

Figure S3: Timescales of mosquito lifespan and parasite development....................7

Methods: Disaggregation of CRU data into hourly rainfall time series....................8

Methods: Summary of data sources....................8

ERA Interim data....................8

Vegetation and soil properties....................9

Table S3: Summary of data sources....................10

Methods: 2080-2099 Precipitation time series....................11

Figure S4: Effect of changing rainfall predictions on *D*, *m*, and *VC*....................12

Results: Alternate *EIP* formulation....................13

Figure S5: Summary of changes to *D*, *m*, and *VC* using alternate *EIP*....................14

References....................15

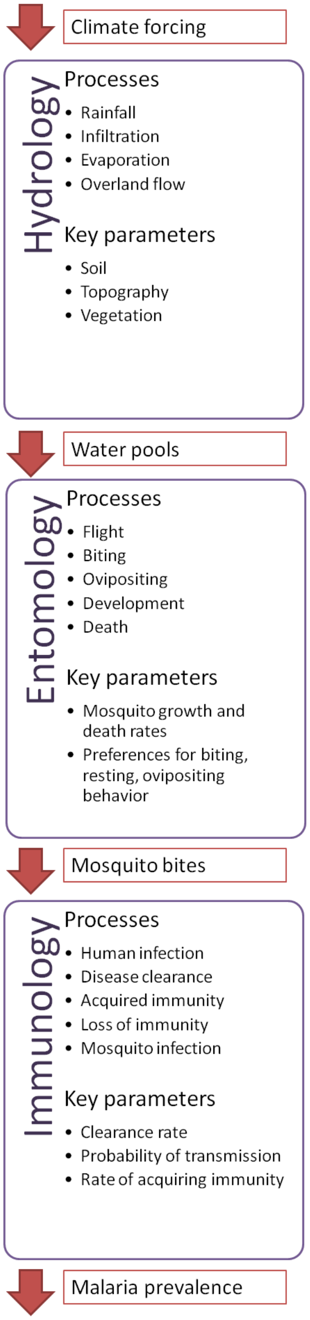

Figure S1: Schematic of HYDREMATS model. This schematic diagram lists the major processes and key parameters represented by the Hydrology, Entomology and Immunology components of HYDREMATS. The arrows represent information that is passed from one component to the next. Model outputs from each component are spatially and temporally explicit. The immunology component is not used in this paper.

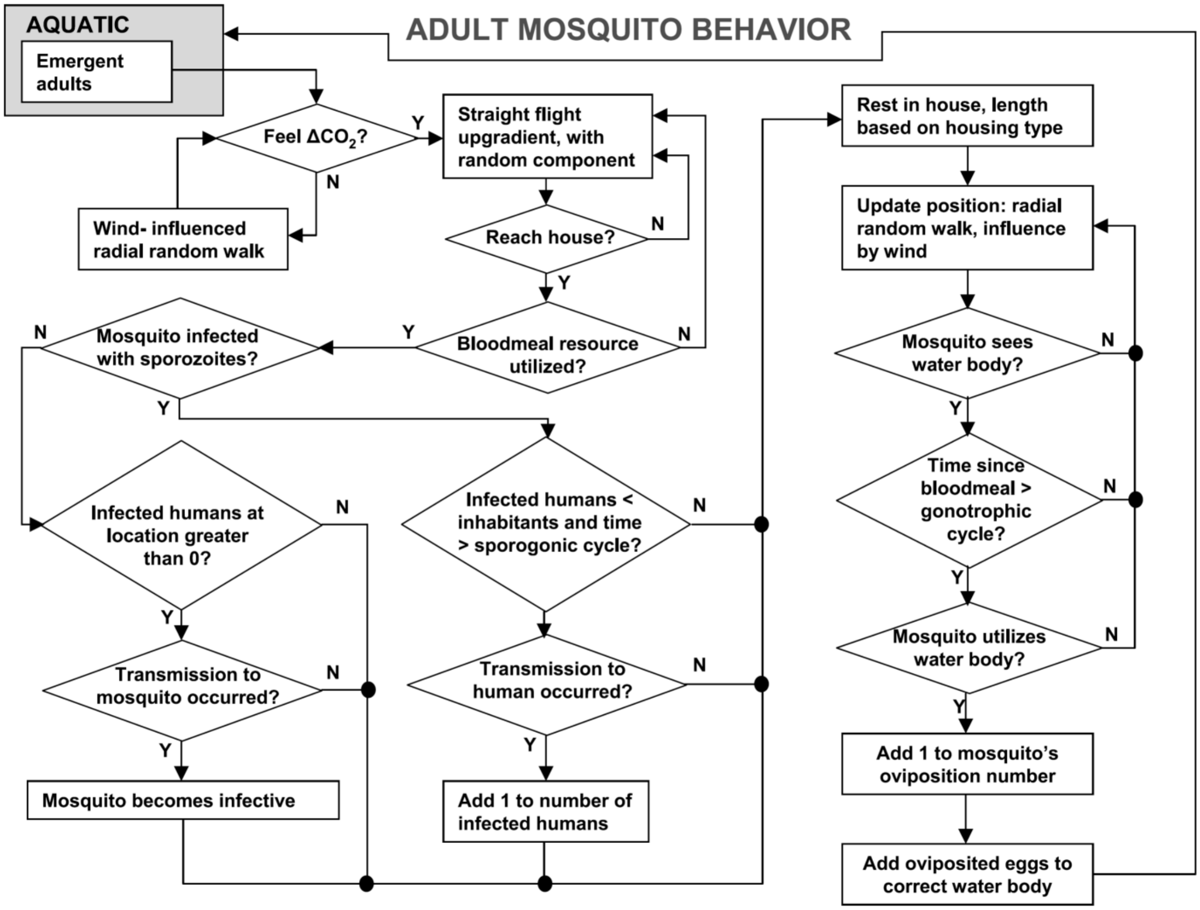

Figure S2 Adult mosquito simulation flow. During each timestep, the model updates each individual mosquito as she progresses through her life cycle. Mosquito attributes are updated as they interact with the environment and human agents. Reprinted from "Hydrology of malaria: Model development and application to a Sahelian village," by Bomblies A, Duchemin JB, Eltahir EAB. 2008. Water Resour Res 44.

Table S1. Parameters used in entomology model.

Variable	nominal value	units	reference	sensitivity[a]
**Aquatic stage simulation**				
cannibalism rate	0.0008	hr^{-1}	Koenraadt and Takken (2003)	medium
number of eggs lain per oviposition	150		Detinova and Gillies (1964)	low
egg death rate	0.001	hr^{-1}	none	very low
weight of first-stage larvae	0.02	mg	none	low
weight of stage 2 larvae	0.16	mg	none	medium
weight of stage 3 larvae	0.3	mg	none	medium
weight of stage 4 larvae	0.45	mg	none	low
lag time for predators to establish	200	hr	Depinay et al. (2004)	low
carrying capacity of pools	100	$mg\ m^{-2}$	Depinay et al. (2004)	low
larvae death rate	0.005	hr^{-1}	Hoshen and Morse (2004)	very low
maximum predation rate for larvae	0.02	hr^{-1}	none	medium
pupae predation rate	0.005	hr^{-1}	none	very low
**Adult mosquito simulation**				
human infection clearing rate	0.0005	hr^{-1}	Hoshen and Morse (2004)	very low
degree days above 18ºC necessary for sporozoites	111	deg-day	Detinova (1962)	very low
probability that a mosquito takes a bloodmeal	0.06		none	low
average mosquito flight velocity	25	$m\ hr^{-1}$	Costantini et al. (1996b)	low
time required for gametocyte development	336	hr	Hoshen and Morse (2004)	very low
weighting of random walk vs. straight line	0.7		none	medium
resting time	48	hr	none	medium
threshold distance for visual cues	15	m	Gillies (1980)	low
utilization probability of water	0.3		none	low

Adapted from "Hydrology of malaria: Model development and application to a Sahelian village," by Bomblies A, Duchemin JB, Eltahir EAB. 2008. Water Resour Res 44.

[a]model sensitivity to parameter values gauged by maximum simulated mosquito abundance

Methods: Development rate of aquatic-stage mosquitoes

The progression of subadult mosquitoes at each hourly timestep is given by the temperature-dependent rate, $r(T_w)$, developed by Depinay et al. (2004) based on enzyme kinetics:

$$r(T_w) = \frac{\rho_{25°C} \cdot \frac{T_W}{298} \cdot exp\left[\frac{\Delta H_A^{\neq}}{R} \cdot \left(\frac{1}{298} - \frac{1}{T_W}\right)\right]}{1 + exp\left[\frac{\Delta H_L}{R} \cdot \left(\frac{1}{T_{0.5L}} - \frac{1}{T_W}\right)\right] + exp\left[\frac{\Delta H_H}{R} \cdot \left(\frac{1}{T_{0.5H}} - \frac{1}{T_W}\right)\right]} \qquad [S1]$$

where T is the mean water temperature over the time interval (1 hour); $\rho_{25°C}$ is the development rate at 25°C given no temperature inactivation of critical enzyme; $\Delta H_A^{\neq}$ is the enthalpy of activation of the catalyzed reaction; ΔH_L and ΔH_H are the enthalpy changes due inactivation of the enzyme from low and high temperature, respectively; $T_{0.5L}$ and $T_{0.5H}$ are the water temperatures at which 50% of the enzyme is inactivated by low and high temperature respectively; and $R=1.987 cal/mol$ is the universal gas constant.

Table S2: Parameters for development rate of subaquatic mosquitoes.

Developmental stage	$\rho_{25°C}$	$\Delta H_A^{\neq}$	ΔH_L	$T_{0.5L}$	ΔH_H	$T_{0.5H}$
**Egg**	0.0413	1	-170644	288.8	1000000	313.3
**Larvae**	0.037	15684	-229902	286.4	822285	310.3
**Pupae**	0.034	1	-154394	313.8	554707	313.8
**Adult**	0.02	1000	-75371	293.1	388691	313.4

Reprinted from "A simulation model of African anopheles ecology and population dynamics for the analysis of malaria transmission," by Depinay JM, Mbogo CM, Killeen G, Knols B, Beier J, Carlson J et al. 2004. *Malaria Journal* 3:29.

Methods: Alternate *EIP* formulation

In our analysis, we follow the majority of current malaria models (Craig et al. 1999; Ermert et al. 2012; Guerra et al. 2008) in using the well-established Detinova (1962) curve for the extrinsic incubation period (*EIP*) of *Plasmodium falciparum* in Anopheles mosquitoes. However, it has been suggested that this curve does not accurately describe *EIP* at high temperatures (Ikemoto 2008; Paaijmans et al. 2009). Observational data of *EIP* at high temperatures are difficult to obtain due to the low rates of mosquito survival. In data compiled by Boyd comparing EIP to temperature (1949), parasite development was possible at temperatures as high as 35°C, but was interrupted at 37°C due to mosquito mortality. Two more recent studies have shown parasite development to be significantly hindered at temperatures above 30°C (Eling et al. 2001; Noden et al. 1995). However a contradictory study found that parasites were able to develop in wild strain mosquitoes at temperature of 30°C and 32°C, though with decreased survival (Okech et al. 2004).

Here, we consider the alternate non-linear curve proposed by Paaijmans (2009). The timescales for the temperature dependent processes for malaria transmission are shown in Figure S3. At temperatures above 30°C, there is a significant difference between the two *EIP* curves; the Detinova curve continues to decrease while the Paaijmans curve increases rapidly. At temperatures above 34°C, the Detinova curve gives an *EIP* of just over 6 days, while the Paaijmans curve indicates that transmission is blocked.

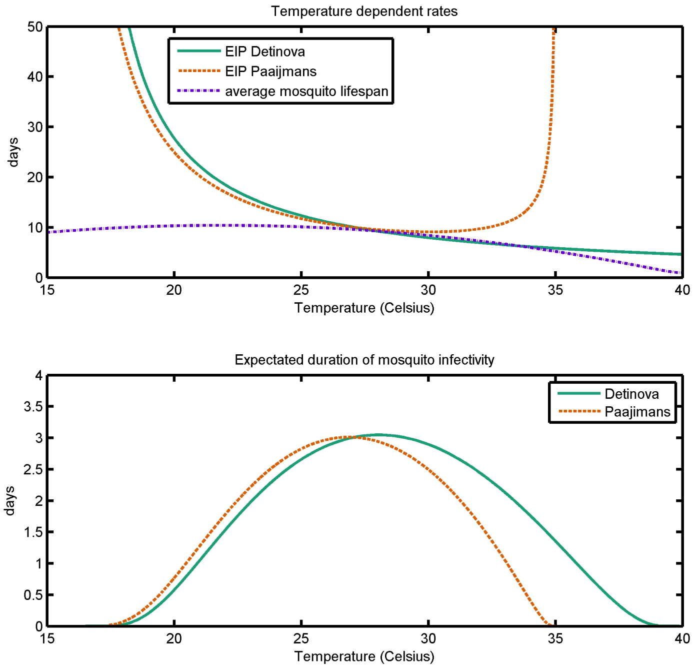

Figure S3: Timescales of mosquito lifespan and parasite development. The two formulations of *EIP* are shown in blue (Detinova) and green (Paaijmans) in the upper plot, and the average mosquito lifespan is shown in red. Estimated values for the duration of infectivity (*D*) according to the two *EIP*s are shown in the lower plot.

Methods: Disaggregation of CRU data into hourly rainfall time series

The environmental processes simulated by HYDREMATS require meteorological inputs at high temporal resolution. The mean baseline rainfall from CRU required disaggregation into hourly resolution before being used as an input. The spatio-temporal dissagregation of rainfall for hydrological applications is a well researched problem, and is often done using various statistical models parameterized by assumed or observed characteristics of finer scale rainfall events (for example Bo et al. 1994; Mackay et al. 2001; Margulis 2001; Segond et al. 2007). Here, we take advantage of high resolution satellite observations of rainfall from the Climate Prediction Center Morphing Technique (CMORPH) data set, which gives ~8km resolution rainfall data every 30 minutes (Joyce et al. 2004). After applying the bias-correction described in Yamana and Eltahir (2011), we use the hourly rainfall observations from CMORPH data at each village to disaggregate baseline CRU rainfall into realistic storm events. The result is an hourly rainfall time series with mean annual rainfall equal to long-term observations from CRU, and patterns of hourly rainfall observations from CMORPH.

Methods: Summary of data sources

ERA Interim data

Temperature, wind speed, wind direction, and radiation data were taken from the ERA Interim data set (Dee et al. 2011) for the grid cell containing each village being simulated; we assume uniform conditions within the 0.75 degree ERA grid cell. ERA Interim data were adjusted for HYDREMATS as follows. Wind speed was brought from 10 m to 2 m by assuming a logarithmic profile. Wind and radiation data were linearly extrapolated from the 3-hour resolution provided by ERA to the 1-hour resolution required by HYDREMATS.

A comparison of the diurnal temperature cycles given by ERA Interim and ground observations at three locations across ecoclimate zones in our study region (Banizoumbou, Niger; Agoufou, Mali; and Djougou, Benin) indicated that while the reanalysis data gave good estimates of the daily mean temperature, the diurnal range was underestimated. The regression coefficients below were calculated using the diurnal temperature ranges of the three ground stations over one year and applied to daily ERA temperature ranges:

$$R_{corrected} = R_{ERA} \times 1.03 + 5.25\text{C}^{\circ}$$

The maximum and minimum daily temperatures were then computed as the daily temperature mean from ERA plus or minus the corrected range divided by two. The hourly temperature was calculated assuming a sinusoidal curve during daylight hours and an exponential decrease between sunset and sunrise, as described in Paaijmans et al.(2009)

Vegetation and soil properties

The dominant vegetation type at each location was obtained from the University of Maryland Land Cover Classification (Hansen et al. 2000). Soil properties were taken from the Harmonized World Soil Database (FAO and ISRIC). A thin layer of low-permeability soil is included in the model to account for soil crusting that occurs throughout West Africa under cultivated conditions (Morin 1993).

Table S3. Summary of data sources.

	Data source	Spatial resolution	Temporal resolution	Reference
**Baseline Climatology**				
temperature	CRU TS 3.1	0.5 x 0.5 degree	1 month	Mitchell and Jones, 2005
rainfall	CRU TS 3.1	0.5 x 0.5 degree	1 month	Mitchell and Jones, 2005
**Meteorological Inputs for HYDREMATS Simulation**				
precipitation	CMORPH	~8km	30 min	Joyce et al., 2004
temperature	ERA-Interim	.75 x .75 degree	3 hour	Dee et al., 2011
wind speed	ERA-Interim	.75 x .75 degree	3 hour	Dee et al., 2011
wind direction	ERA-Interim	.75 x .75 degree	3 hour	Dee et al., 2011
surface radiation	ERA-Interim	.75 x .75 degree	3 hour	Dee et al., 2011
**Other HYDREMATS inputs**				
soil type	HWSD	~1km		FAO, 2009
vegetation	UMD landcover	1km		Hansen et al., 2000
topography	Computed from Envisat synthetic aperture radar and ground survey	10 m		Toutin and Gray, 2000; Bomblies et al. 2008
household locations	Quickbird image	0.6 m		Bomblies et al. 2008
**Climate predictions**				
Rainfall and temperature anomalies 2080-2099	Various climate models (See Table 2) from IPCC AR4 emissions scenario A1B	Range from 1.4 x 1.4 degree to 3.9 x 3.9 degree	1 month	IPCC, 2011

Methods: 2080-2099 Precipitation time series

We assume that climate change will take the form of shifts of the rainfall gradient, based on the observation that historical changes in rainfall regimes in this region took the form of northward or southward shifts of the rainfall gradient (Bomblies and Eltahir 2010; Irizarry-Ortiz et al. 2003). The 2080-2099 precipitation time series were created by selecting a grid cell directly north (for decreased rainfall scenarios) or south (for increased rainfall scenarios) of each site where rainfall is currently equal to the annual rainfall predicted by a GCM for 2080-2099. For example, the village selected to represent Zone 2 is located at 15.05N, 8.33E, where the baseline rainfall is 259 mm/year. Applying the predicted changes from the GCMs, the dry scenario should have an average rainfall of 53 mm/year and the wet scenario should have an average rainfall of 366 mm/year. The rainfall time series for the wet scenario comes from a location south of our village, at 14.25N, 8.33E where current rainfall averages approximately 366 mm/year. Rainfall inputs for the dry scenario come from a location north of our village at 19.25N, 8.33E where current rainfall averages 54 mm/year. We again disaggregate the coarse resolution rainfall data by applying the hourly patterns of rainfall observed by CMORPH.

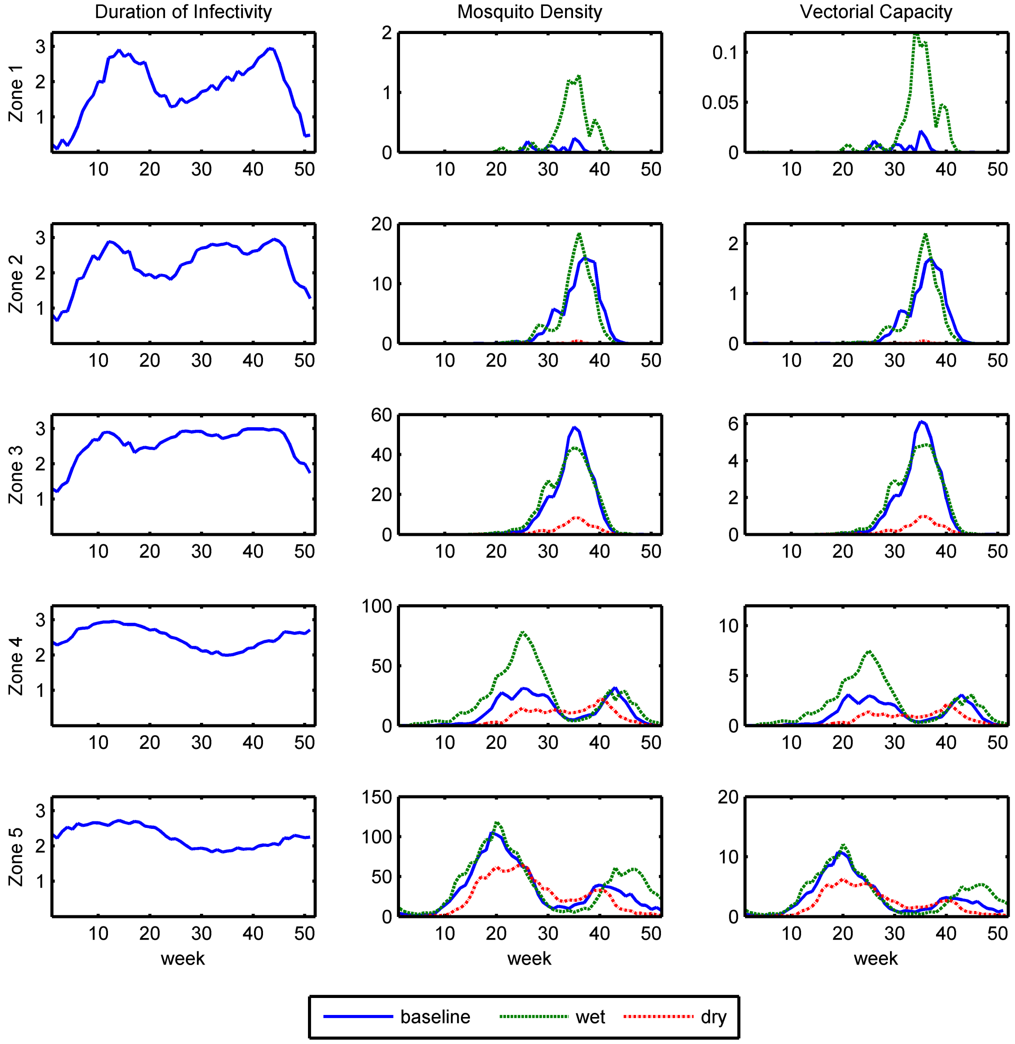

Figure S4. Estimated effect of changing rainfall predictions on *D*, *m*, and *VC*. Weekly average values based on simulations for *D* (the duration of infective life, column 1, in days), *m* (mosquito density, column 2, the number of mosquitoes per human), and *VC* (vectorial capacity, column 3, the average number of human inoculations of a parasite originating from a single case of malaria if all vectors biting the original case became infected) as a result of changes in rainfall only, from Zone 1 (top row) through Zone 5 (bottom row). Simulation-based estimates at baseline (1980-1999) and for 2080-2099 according to the wet and dry climate change scenarios for each zone are shown in blue, green, and red, respectively.

Results: Alternate *EIP* formulation

Simulations using the alternate *EIP* (Paaijmans 2009) generally had lower *D,* and thus lower *VC*, throughout West Africa, with the effect being most pronounced in the hottest regions (Zones 1 and 2). While the magnitudes of the values are lowered, the overall observations in terms of seasonality, difference between zones, and differences between current and future climates remain largely unchanged.

When we average the changes over the seven-year simulation period, shown in Figure S4, the results are again very similar to our original findings. The main differences are in the dry-hot scenarios in Zones 4 and 5; while the warming leads to slight increases in vectorial capacity using Detinova equation for *EIP*, there is a slight decrease in *VC* using the Paaijmans equation.

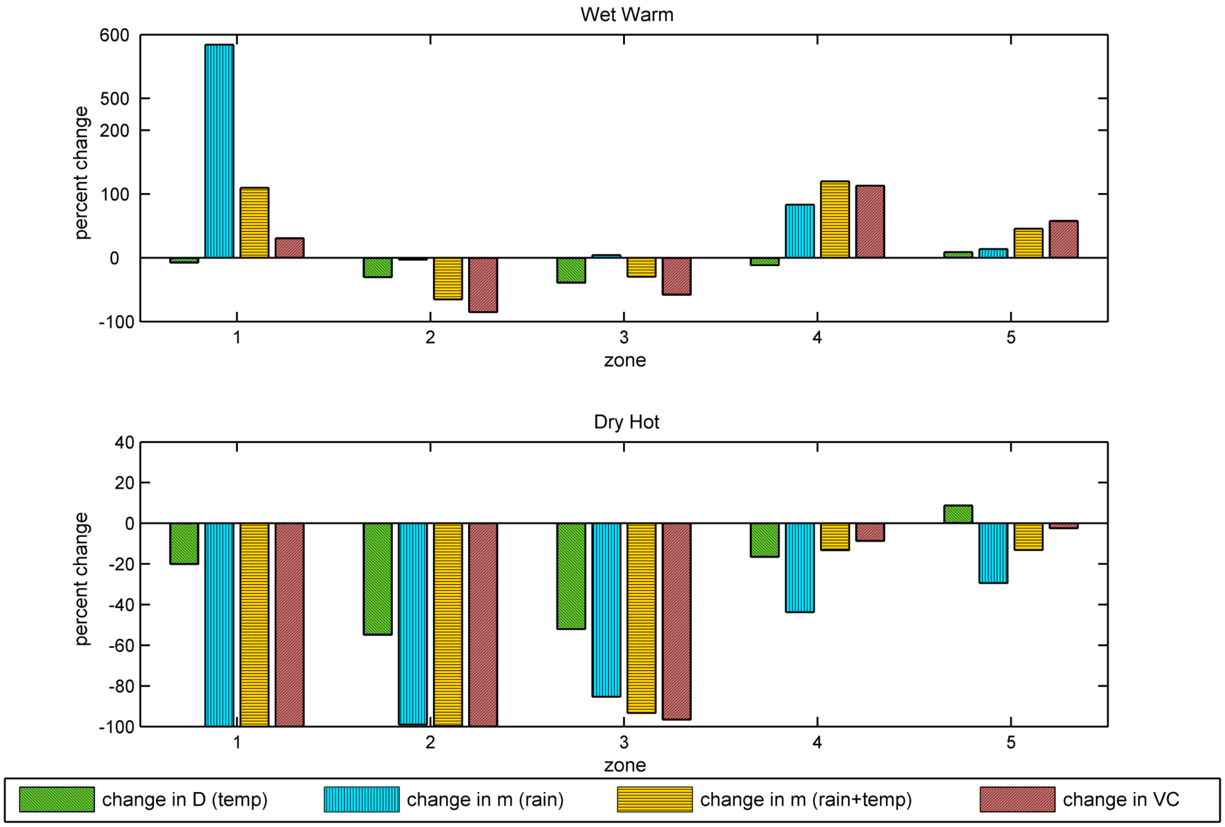

Figure S5: Summary of changes to *D*, *m*, and *VC* using the alternate *EIP*. Mean changes in vectorial capacity over 7-year simulations for the wet-warm (top) and dry-hot (bottom) climate change scenarios for climatic zones 1 through 5 (Sahelo-Saharan to Guinea Coast respectively) using the Paaijmans (2009) equation for *EIP*. Note the abbreviated vertical axis in the top figure.

References

Bomblies A, Duchemin JB, Eltahir EAB. 2008. Hydrology of malaria: Model development and application to a sahelian village. Water Resour Res 44.

Bomblies A, Eltahir EAB. 2010. Assessment of the impact of climate shifts on malaria transmission in the sahel. EcoHealth:1-12.

Bo Z, Islam S, Eltahir E. 1994. Aggregation-disaggregation properties of a stochastic rainfall model. Water Resour Res 30(12):3423-3435. Boyd MF. 1949. Epidemiology: Factors related to the definitive host. Malariology 1:608-697.

Costantini C, LI S, Torre AD, Sagnon N, Coluzzi M, Taylor CE. 1996. Density, survival and dispersal of anopheles gambiae complex mosquitoes in a west african sudan savanna village. Med Vet Entomol 10(3):203-219.

Craig MH, Snow RW, le Sueur D. 1999. A climate-based distribution model of malaria transmission in sub-Saharan Africa. Parasitology Today 15(3):105-111; doi: DOI: 10.1016/S0169-4758(99)01396-4.

Dee DP, Uppala SM, Simmons AJ, Berrisford P, Poli P, Kobayashi S et al. 2011. The ERA-interim reanalysis: Configuration and performance of the data assimilation system. Q J R Meteorol Soc 137(656):553-597; doi: 10.1002/qj.828.

Depinay JM, Mbogo CM, Killeen G, Knols B, Beier J, Carlson J et al. 2004. A simulation model of african anopheles ecology and population dynamics for the analysis of malaria transmission. Malar J 3:29; doi: 10.1186/1475-2875-3-29.

Detinova TS. 1962. Age-grouping methods in diptera of medical importance with special reference to some vectors of malaria. Monogr Ser World Health Organ 47:13-191.

Detinova T, Gillies M. 1964. Observations on the determination of the age composition and epidemiological importance of populations of anopheles gambiae giles and anopheles funestus giles in tanganyika. Bull World Health Organ 30(1):23.

Eling W, Hooghof J, van de Vegte-Bolmer M, Sauerwein R, Van Gemert G. 2001. Tropical temperatures can inhibit development of the human malaria parasite plasmodium falciparum in the mosquito. In: Proceedings of the Section Experimental and Applied Entomology - Netherlands Entomological Society, 151-156.

Ermert V, Fink AH, Morse AP, Paeth H. 2012. The impact of regional climate change on malaria risk due to greenhouse forcing and land-use changes in tropical Africa. Environ Health Perspect 120(1):77.

FAO (Food and Agriculture Organization). 2009. Harmonized World Soil Database v1.1. Available: http://www.fao.org/nr/land/soils/harmonized-world-soil-database/en/ [accessed 31 Jul 2012].

Gillies M. 1980. The role of carbon dioxide in host-finding by mosquitoes (diptera: Culicidae): A review. Bull Entomol Res 70:525-532.

Guerra CA, Gikandi PW, Tatem AJ, Noor AM, Smith DL, Hay SI et al. 2008. The limits and intensity of plasmodium falciparum transmission: Implications for malaria control and elimination worldwide. PLoS Med 5(2):e38.

Hansen M, DeFries R, Townshend J, Sohlberg R. 2000. Global land cover classification at 1 km spatial resolution using a classification tree approach. Int J Remote Sens 21(6):1331-1364.

Hoshen M, Morse A. 2004. A weather-driven model of malaria transmission. Malaria Journal 3(1):32.

Ikemoto T. 2008. Tropical malaria does not mean hot environments. J Med Entomol 45(6):963-969.

IPCC (Intergovernmental Panel on Climate Change). 2011. Model output described in the 2007 IPCC Fourth Assessment Report (SRES scenarios), multi-year means. Available: http://www.ipcc-data.org/ar4/gcm_data.html [accessed 14 Feb 2013].

Joyce RJ, Janowiak JE, Arkin PA, Xie P. 2004. CMORPH: A method that produces global precipitation estimates from passive microwave and infrared data at high spatial and temporal resolution. J Hydrometeorol 5(3):487-503.

Koenraadt CJM, Paaijmans KP, Githeko AK, Knols BGJ, Takken W. 2003. Egg hatching, larval movement and larval survival of the malaria vector anopheles gambiae in desiccating habitats. Malaria Journal 2(1):20.

Mackay N, Chandler R, Onof C, Wheater H. 2001. Disaggregation of spatial rainfall fields for hydrological modelling. Hydrology and Earth System Sciences Discussions 5(2):165-173.

Margulis SA, Entekhabi D. 2001. Temporal disaggregation of satellite-derived monthly precipitation estimates and the resulting propagation of error in partitioning of water at the land surface. Hydrology and Earth System Sciences 5:27-38.

Mitchell TD, Jones PD. 2005. An improved method of constructing a database of monthly climate observations and associated high-resolution grids. Int J Climatol 25(6):693-712; doi: 10.1002/joc.1181.

Morin J. 1993. Soil crusting and sealing. Soil tillage in Africa: Needs and challenges. FAO Soils Bulletin (FAO) 69:41-41-67.

NOAA (National Oceanic and Atmospheric Administration). 2009. Ferret User's Guide. Available: http://ferret.pmel.noaa.gov/Ferret/documentation/users-guide [accessed 2 Feb 2013]

Noden B, Kent M, Beier J. 1995. The impact of variations in temperature on early plasmodium falciparum development in anopheles stephensi. Parasitology 111(05):539-545.

Okech BA, Gouagna LC, Kabiru EW, Walczak E, Beier JC, Yan G et al. 2004. Resistance of early midgut stages of natural plasmodium falciparum parasites to high temperatures in experimentally infected anopheles gambiae (diptera: Culicidae). J Parasitol 90(4):764-768.

Paaijmans KP, Read AF, Thomas MB. 2009. Understanding the link between malaria risk and climate. Proceedings of the National Academy of Sciences 106(33):13844.

Segond M, Neokleous N, Makropoulos C, Onof C, Maksimovic C. 2007. Simulation and spatio-temporal disaggregation of multi-site rainfall data for urban drainage applications. Hydrological Sciences Journal 52(5):917-935.

Toutin T, Gray L. 2000. State-of-the-art of elevation extraction from satellite SAR data. ISPRS Journal of Photogrammetry and Remote Sensing 55(1):13-33.

Yamana TK, Eltahir EAB. 2011. On the use of satellite-based estimates of rainfall temporal distribution to simulate the potential for malaria transmission in rural Africa. Water Resour Res 47(2):W02540.
